# High‐efficiency transformation of archaea by direct PCR products with its application to directed evolution of a thermostable enzyme

**DOI:** 10.1111/1751-7915.13613

**Published:** 2020-06-29

**Authors:** Yunhong Song, Zhiguang Zhu, Wei Zhou, Yi‐Heng P. Job Zhang

**Affiliations:** ^1^ Tianjin Institute of Industrial Biotechnology Chinese Academy of Sciences 32 West 7th Avenue Tianjin Airport Economic Area Tianjin 300308 China

## Abstract

Hyperthermophilic archaea with unique biochemical and physiological characteristics are important organisms for fundamental research of life science and have great potential for biotechnological applications. However, low transformation efficiency of foreign DNA molecules impedes developments in genetic modification tools and industrial applications. In this study, we applied prolonged overlap extension PCR (POE‐PCR) to generate multimeric DNA molecules and then transformed them into two hyperthermophilic archaea, *Thermococcus kodakarensis* KOD1 and *Pyrococcus yayanosii* A1. This study was the first example to demonstrate the enhanced transformation efficiencies of POE‐PCR products by a factor of approximately 100 for *T. kodakarensis* KOD1 and 8 for *P. yayanosii* A1, respectively, relative to circular shuttle plasmids. Furthermore, directed evolution of a modestly thermophilic enzyme, *Methanothermococcus okinawensis* 3‐hydroxy‐3‐methylglutaryl coenzyme A reductase (HMGR), was conducted to obtain more stable ones due to high transformation efficiency of *T. kodakarensis* (i.e. ~3 × 10^4^ CFU per μg DNA). *T. kodakarensis* harbouring the most thermostable MoHMGR mutant can grow in the presence of a thermostable antibiotic simvastatin at 85°C and even higher temperatures. This high transformation efficiency technique could not only help develop more hyperthermophilic enzyme mutants via directed evolution but also simplify genetical modification of archaea, which could be novel hosts for industrial biotechnology.

## Graphical abstract

We developed a very simple technology to transfer prolonged overlap extension PCR (POE‐PCR) products to two hyperthermophilic archaea with enhanced transformation efficiencies. Via it, we can evolve mesophilic enzymes to hyperthermophilic ones by directed evolution.

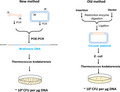

## Introduction

Archaea is one of the three domains of organisms. Hyperthermophilic archaea with their optimal growth temperatures of more than 70°C may be the most primitive extant forms of the life. Many of these organisms have unique biochemical and physiological characteristics with important biotechnological implications (Straub *et al*., 2018). They could be valuable thermophilic microbial cell factories and invaluable sources of thermostable enzymes. However, their application has been largely hampered because of a lack of facile and high transformation efficiency genetic modification tools (Zeldes *et al*., 2015; Straub *et al*., 2018). Some transformation approaches, such as CaCl_2_ incubation, heat shock and electroporation, have been established with a few hyperthermophilic archaea, such as *Thermococcus kodakarensis* KOD1 (Hileman and Santangelo, 2012), *Pyrococcus furiosus* COM1 (Lipscomb *et al*., 2011) and several *Sulfolobus* species (Peng *et al*., 2017) (Table S1). However, low transformation efficiencies and long preparation time of shuttle plasmids hinder the development of robust and efficient genetic systems and other applications, except for *Sulfolobus* species (Berkner *et al*., 2007; Jaubert *et al*., 2013) and a high‐competent *P. furiosus* mutant (Lipscomb *et al*., 2011).

*Thermococcus kodakarensis* KOD1 is an anaerobic, heterotrophic euryarchaeon with an optimal growth temperature of ~ 85°C. It grows on a few carbon sources, such as peptides, starch, chitin or pyruvate, with S˚ or H^+^ as an electron acceptor for generating H_2_S or H_2_, respectively (Atomi *et al*., 2004). This microorganism grows rapidly to relatively high cell densities of ∼ 10^9^ cells ml^−1^ with a doubling time of 40 min (Atomi *et al*., 2004). The transformed colonies in solid plates can be selected based on an auxotrophic marker (i.e. tryptophan, arginine, uracil or agmatine) or antibiotic resistance (i.e. mevinolin or simvastatin) (Hileman and Santangelo, 2012). Now, *T. kodakarensis* is a model archaeon for in‐depth understanding of physiology and the origin of life of archaea, the discovery of novel metabolic pathways (Santangelo *et al*., 2011; Aslam *et al*., 2017) and thermophilic enzymes (Elshawadfy *et al*., 2014; Zhang and Tripathi, 2017; Okano *et al*., 2018), and biotechnological applications for the expression of complicated enzyme complexes, such as soluble [NiFe] hydrogenase (Song et al., 2018b). Although it is naturally competent for the uptake of either linear or circular DNA (Sato *et al*., 2005), its low transformation efficiency of 10^1^–10^2^ transformants per μg of (circular) plasmid DNA still limits its wide adoption (Hileman and Santangelo, 2012; Straub *et al*., 2018).

*Pyrococcus yayanosii* A1 is a strictly anaerobic and facultative piezophilic hyperthermophile (Li *et al*., 2015). Its ranges of growth temperature and pressure are from 80 to 108°C (optimal 98°C) and 20 to 120 MPa (optimal 52 MPa), respectively. This strain is valuable in the study of functional and comparative genomics studies with in‐depth understanding of the piezophilic adaptation. However, its transformation efficiency was as low as 10^2^ transformants per μg of pLMO01 plasmid DNA (Li *et al*., 2015).

Different microorganisms have different DNA uptake mechanisms (van Wolferen *et al*., 2016; Wagner *et al*., 2017; Pimentel and Zhang, 2018). Three foreign DNA uptake mechanisms of archaea and bacteria are natural transformation, conjugation and transduction (van Wolferen *et al*., 2013; Wagner *et al*., 2017). To our limited knowledge, approximately 80 naturally competent bacteria and only five archaea have been reported to take up DNA molecules from the external environment (Chen and Dubnau, 2004; Kruger and Stingl, 2011; Wagner *et al*., 2017). Bacterial DNA uptake mechanisms are well understood at the molecular level. Gram‐positive bacteria *Bacillus* spp. prefers actively taking up double‐stranded DNA molecules to linear DNA molecules because one DNA strand is degraded and the other strand is transported actively across the membrane and then is cyclized to a circular plasmid while circular plasmid DNA is not a substrate for this active DNA uptake process (Zhang and Zhang, 2011; You *et al*., 2012; Cao *et al*., 2014). In contrast, Gram‐negative *E. coli* strains assimilate the closed circular plasmid through a weakened cell envelope and have a weak ability to cyclize the double‐stranded DNA to a circular plasmid by recombineering (Symington *et al*., 1985; Sauer and Henderson, 1988). However, little knowledge is available for archaea (van Wolferen *et al*., 2016; Wagner *et al*., 2017). Both *T. kodakarensis* KOD1 and *P. yayanosii* A1 are among natural competent ones. However, the natural transformation mechanisms in archaea have still not been elucidated on a molecular level; for example, homologs related to bacterial competence were not identified in archaeal species (Wagner *et al*., 2017).

A simple PCR technique – prolonged overlap extension PCR (POE‐PCR) – has been developed to generate multimeric DNA molecules having extra‐large molecular weights (Zhang and Zhang, 2011; You *et al*., 2012). The POE‐PCR products were prepared based on two PCR products containing ~ 50 bp overlapped sequences at both termini, and the multimeric PCR products were formed by using prolonged time of the PCR reaction, wherein both PCR products function as both primers and DNA templates. For *B. subtilis* and related *Bacillus* spp. (Zhang and Zhang, 2011; You *et al*., 2012; Liu *et al*., 2013; Cao *et al*., 2014; Fu *et al*., 2018), the transformation of POE‐PCR products has several orders of magnitude higher efficiencies than those of circular plasmids or linear double‐stranded DNA (Contente and Dubnau, 1979; Mottes *et al*., 1979; Zhang and Zhang, 2011). Although it prefers circular plasmid, *E. coli* is able to be transformed by POE‐PCR product (You *et al*., 2012). To our limited knowledge, no one has attempted to test whether archaea are able to take up the multimeric DNA molecules made by POE‐PCR directly.

Besides natural treasure troves of thermostable enzymes (Atomi *et al*., 2011; Rollin *et al*., 2015; Honda *et al*., 2017; You *et al*., 2017; Kim *et al*., 2018), hyperthermophilic microorganisms could also be an ideal host organism for evolving mesophilic or moderately thermophilic enzymes into their thermostable counterparts. Directed evolution is a powerful tool for protein engineering without the detailed understanding of catalytic mechanism and structure information (Giver *et al*., 1998; Schwab and Sterner, 2011; Zhou *et al*., 2018). To our limited knowledge, the only hyperthermophilic microorganism *S. solfataricus* has been demonstrated for directed evolution of mesophilic enzymes to more thermostable ones (Cannio *et al*., 2001).

In this study, we tested the feasibility of transforming POE‐PCR products (i.e. multimeric DNA) to two archaea (i.e. *T. kodakarensis* KOD1 and *P. yayanosii* A1) and obtained much higher transformation efficiencies relative to circular plasmids. Furthermore, we demonstrated a selection‐based strategy for directed evolution of *Methanothermococcus okinawensis* 3‐hydroxy‐3‐methylglutaryl coenzyme A reductase (HMGR) based on *T. kodakarensis*.

## Results

### Multimeric DNA generation

**Fig. 1:**
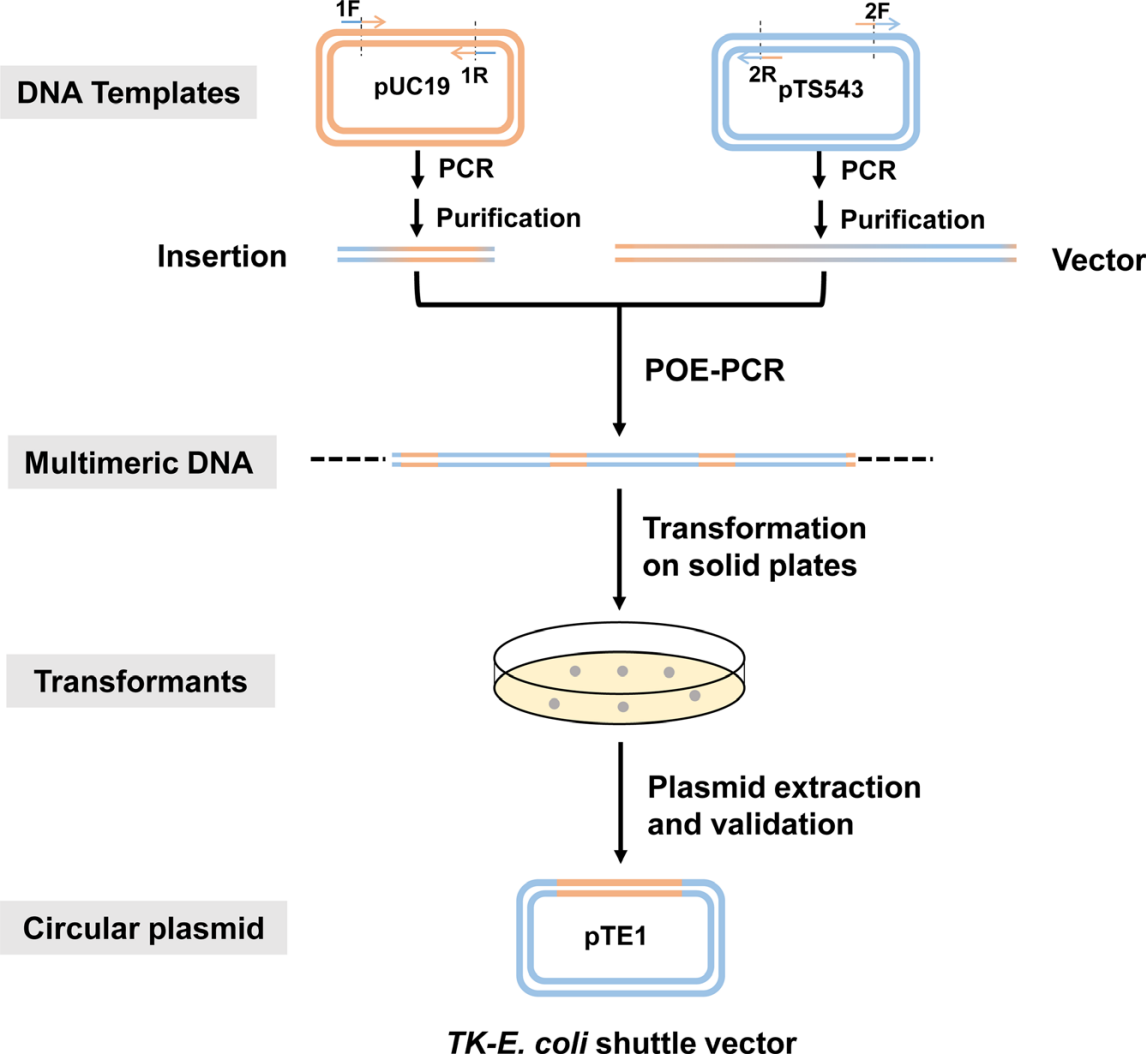
Flow scheme of prolonged overlap extension PCR (POE‐PCR) for the generation of a circular plasmid in *T. kodakarensis*, where the POE‐PCR product (multimeric DNA) was formed by POE‐PCR *in vitro*.

Multimeric DNA molecules can be prepared by POE‐PCR (Fig. 1). First, the linear insertion double‐stranded DNA fragment and the linear double‐stranded DNA vector backbone, both of which contained 3′ and 5′ 40‐ to 50‐bp overlapping termini, were generated by high‐fidelity PCR. Second, the PCR products of the insertion DNA fragment and vector backbone were purified by PCR purification kit. Third, high‐concentration insertion DNA fragment and vector backbone as both primers and templates of PCR can be assembled by POE‐PCR, wherein the PCR extension time was prolonged longer than regular PCR. As a result, the insertion fragment and vector backbone can form multimeric DNA, which were comprised of repetitive plasmid sequences containing the insertion DNA and vector fragments. Last, the POE‐PCR product (without purification) can be transferred into a competent archaeon, which could tailor very large molecular weight multimeric DNA molecules to the circular plasmid that can be duplicated in the host. The cyclization of the concatenated PCR product into a circular plasmid has been demonstrated for Gram‐positive bacteria, such as *B. subtilis*, and Gram‐negative bacteria, such as *E. coli*. This method did not require restriction enzymes, ligase, and was DNA sequence independent. The insertion sequence could have no any unnecessary base pairs (e.g. by using restriction enzyme and ligase) in the resulting plasmid and be inserted into any place of the plasmid. But this method has not been tested in archaea.

### Transformation of T. kodakarensis

**Fig. 2:**
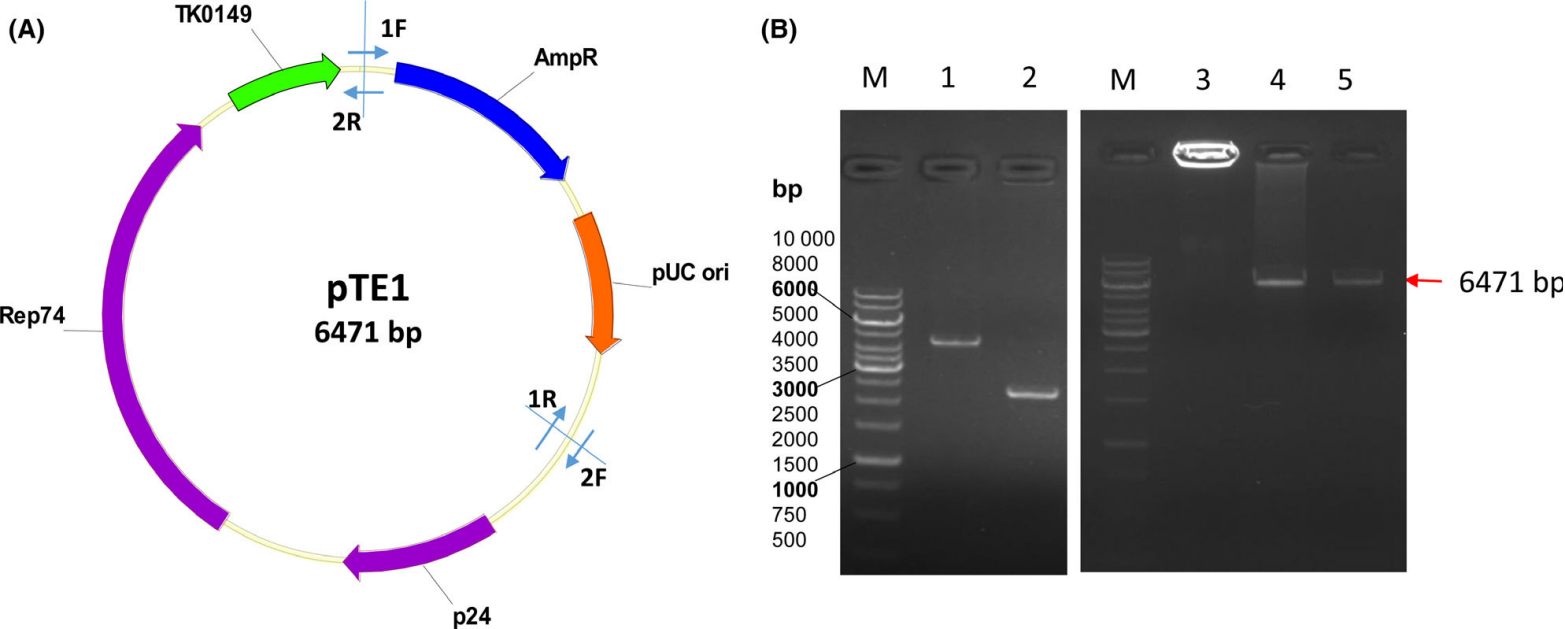
Construction and validation of the *Tk‐Ec* shuttle vector. A. Map of the *Tk‐Ec* shuttle vector. Primers 1F/1R were used to amplify the inserted DNA fragment from pUC19, which contained the autonomous replication region of *E. coli*, and primers 2F/2R were used to amplify the vector backbone from pTS543, which contained the autonomous replication of *TK*. B. Agarose analysis of the POE‐PCR products and the resulting plasmid. M, DNA ladder markers; lane 1, the PCR product based on 1F/1R; lane 2, the PCR product based on 2F/2R; lane 3, the POE‐PCR product; lane 4, the POE‐PCR product digested with EcoRI; and lane 5, the plasmid extracted from *E. coli* Top10 was digested with EcoRI.

A *T. kodakarensis*‐*E. coli* (Tk‐Ec) shuttle vector pTE1 (Song et al., 2018a,b) was used to transform into *T. kodakarensis* TS559 (Fig. 2A). The insertion DNA fragment and backbone vector were amplified by high‐fidelity PCR (Lanes 1 and 2, Fig. 2B). They were assembled and amplified by POE‐PCR, resulting in the multimeric DNA molecules, which were too large to migrate outside the sampling well (Lane 3). Enzymatic digestion of the POE‐PCR product by a restriction enzyme EcoRI led to a single DNA band, which had the same size of the desired circular plasmid (Lane 4). This POE‐PCR product was used to introduce into strain TS559 directly, and the transformants were then spread on the solid Petri plates. The transformation efficiency of this multimeric DNA was more than 10^3^ transformants per μg of POE‐PCR product (based on DNA), much higher than that of the circular DNA plasmid (e.g. ~100 transformants per μg DNA) (Santangelo *et al*., 2008, 2010). Seven randomly chosen colonies were validated by colony PCR, suggesting that all of the transformants contained the shuttle vector pTE1 (Fig. S1). One of the shuttle plasmids, which was purified from *E. coli* TOP10, was digested by EcoRI, yielding a linear fragment (6471 bp, Lane 5), which was in good agreement with the single band of enzymatic digestion of multimeric DNA (Lane 4). The sequence of plasmid pTE1 was validated by DNA sequencing. These results suggested that the POE‐PCR product (i.e. multimeric DNA) can be used to transform *T. kodakarensis* directly with high efficiencies.

As a control, the transformation a mixture of two linear double‐stranded PCR products (the insertion and vector backbone) cannot lead to any positive colonies (data not shown).

### Optimization of *T. kodakarensis* transformation

**Fig. 3:**
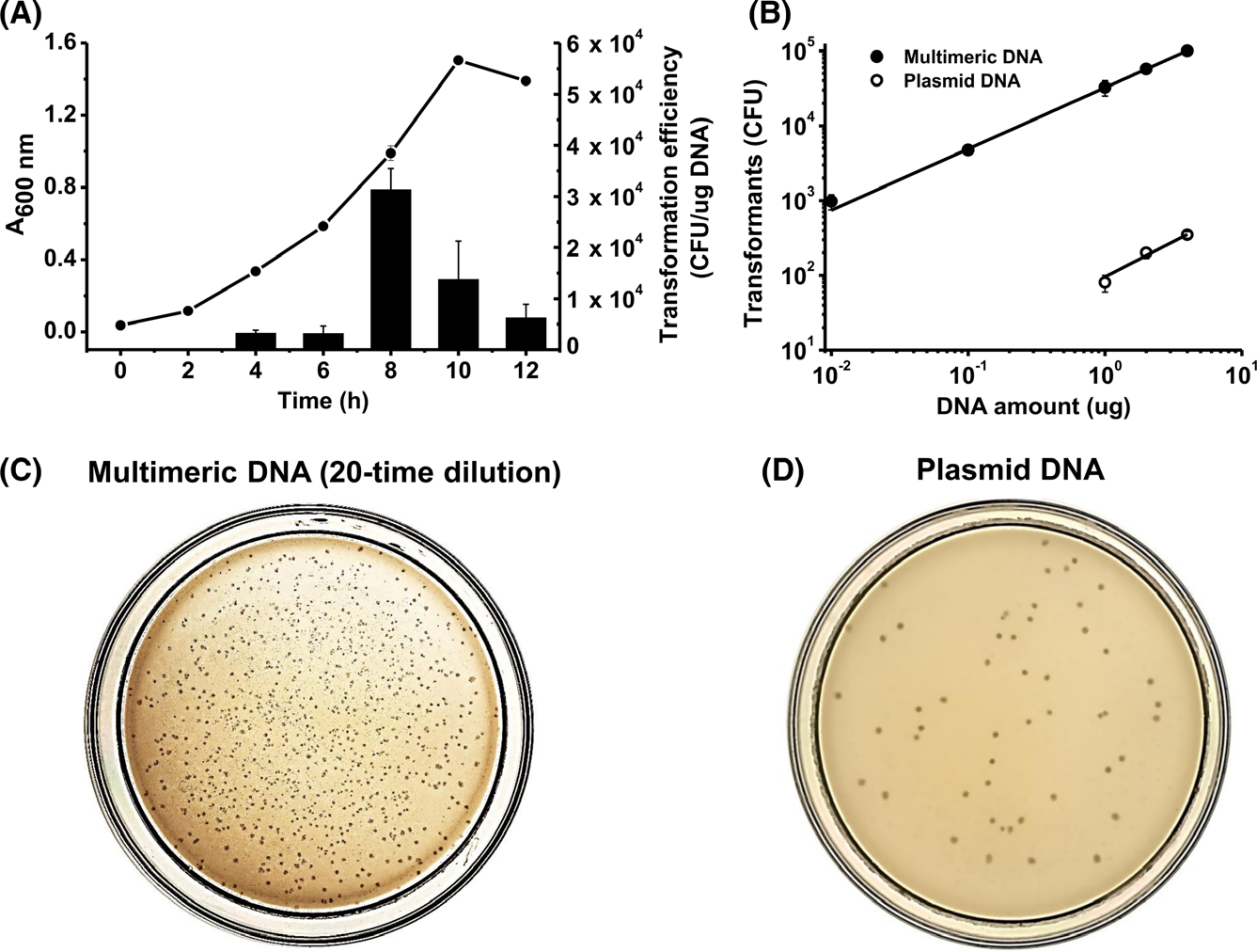
Optimization of *T. kodakarensis* transformation. A. Transformation efficiencies for cells harvested at various growth stages. The growth curve was indicated with a line, and transformation efficiency was indicated with columns. Mean values with standard deviations (error bars) are shown from at least three repeated experiments. B. Relationship of transformation in terms of the DNA amount. Mean values with standard deviations (error bars) from at least three repeats are shown. C. Image of the transformants growing on the plate after the transformation of 1 μg of the multimeric DNA followed by a 20‐time dilution. D. Image of the transformants growing on the plate after the transformation of 1 μg of circular plasmid DNA.

The physiological phases of *T. kodakarensis* could influence its transformation efficiency. The TS559 cells growing at different stages of cell cultures were harvested. The same number of cells was transformed with 100 ng of multimeric DNA (Fig. 3A). The highest transformation efficiency of ~ 3×10^4^ CFU per μg DNA was obtained with cells growing at a mid‐log phase.

We compared the transformation efficiencies of the POE‐PCR product and circular plasmid pTE1 purified from *E. coli* at various concentrations of DNA on the competent cells growing at the mid‐log phase (Fig. 3B). The transformation efficiency of plasmid pTE1 was approximately 10^2^ colony‐forming units (CFUs) per μg of DNA, in good agreement with literature data (Sato *et al*., 2005). In contrast, the transformation efficiency of the POE‐PCR product was over 10^4^ CFUs per μg of DNA. Clearly, the POE‐PCR product enhanced the transformation efficiency of *T. kodakarensis* relative to the plasmid by a factor of 100 (Fig. 3B). The colony number in the solid Petri plates clearly shows that the *T. kodakarensis* prefers transformation with the POE‐PCR product (Fig. 3C) to the circular plasmid (Fig. 3D).

### Directed evolution of MoHMGR in *T. kodakarensis*

**Fig. 4:**
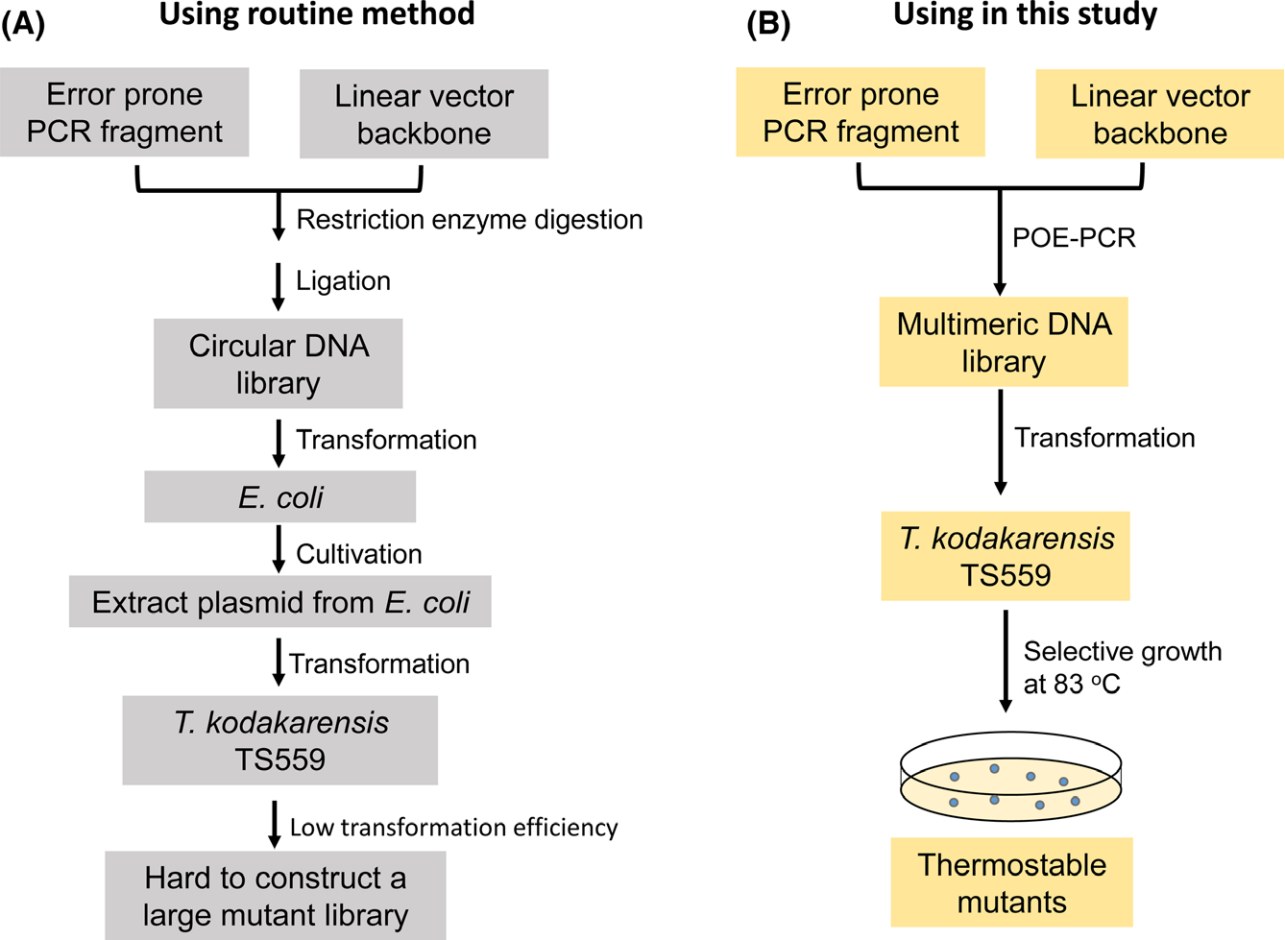
Comparison of the routine method and the new method used in this study for directed evolution in *T. kodakarensis*. A. Routine method for directed evolution in *T. kodakarensis* through *E. coli*. B. Schematic presentation of directed evolution for the more thermostable HMGR mutants. Thermostable mutants were selected at 83°C on solid plates containing 2 μM simvastatin (Sim) without agmatine.

Because of low transformation efficiency of circular plasmids to *T. kodakarensis*, the routine protocol of directed evolution requires a large amount of circular plasmid‐containing mutants which were prepared and purified from *E. coli*. This protocol was time‐consuming and required relatively high laboratory skills (Fig. 4A). Because the multimeric DNA had much higher transformation efficiency for *T. kodakarensis* (Fig. 3), we can simplify the protocol of directed evolution greatly (Fig. 4B).

Simvastatin is a hypolipidaemic thermostable compound impairing the lipid biosynthesis by competitively inhibiting HMG‐CoA reductase (Matsumi *et al*., 2007), and *T. kodakarensis* is sensitive to simvastatin. Therefore, *T. kodakarensis* cannot grow in the presence of simvastatin. To test the feasibility of a new hyperthermophilic host *T. kodakarensis* for directed evolution, we chose a modestly thermophilic enzyme HMGR from *M. okinawensis*, which was not stable at 83°C. The wild‐type *M. okinawensis* HMGR cannot enable *T. kodakarensis* TS559 to grow in the presence of 2 μM simvastatin at 83°C. If more thermostable MoHMGR mutants were obtained by mutagenesis, the strains harbouring more thermostable mutants could grow in the presence of simvastatin at 83°C.

The gene encoding MoHMGR was randomly mutated by error‐prone PCR. The *Mohmgr* mutants were assembled with the backbone of the shuttle vector pTE1 by POE‐PCR, wherein the *Mohmgr* gene was under control of a constitutive Pgdh promoter (Fig. S2). The resulting POE‐PCR product which contained the *Mohmgr* mutant library was transformed into strain TS559. When 1 μl of the POE‐PCR product was used, approximately 10^3^ colonies were formed on the agmatine‐free solid plates. Therefore, 50 μl of POE‐PCR product provided a large‐size mutant library of more than 5 × 10^4^ transformants, sufficient for directed evolution.

**Fig. 5:**
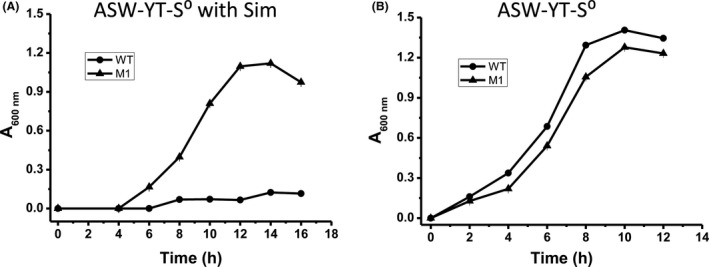
Growth profiles of *T. kodakarensis* expressed thermostable HMGR mutant M1 and wild‐type HMGR WT in the ASW‐YT‐S^0^ medium with Sim (A) and without Sim (B) at 85°C. Mean values with standard deviations (error bars) from at least three repeats are shown.

Via this selection method, a thermostable mutant M1 was identified (Data S2). This mutant contained three amino acid mutations S287C/M322V/V379A (Data S3). The strain harbouring a plasmid encoding the *Mohmgr* mutant M1 grew well in the liquid ASW‐YT‐S⁰ medium containing 4 μM simvastatin at 85°C, while strain harbouring the plasmid containing the wild‐type *Mohmgr* gene failed to grow (Fig. 5A). Both strains showed similar growth patterns in the liquid ASW‐YT‐S⁰ medium without simvastatin (Fig. 5B). Therefore, we validated the technical feasibility of the use of *T. kodakarensis* as a host for directed evolution with direct transformation of the POE‐PCR products.

### Transformation of *P. yayanosii* A1

**Fig. 6:**
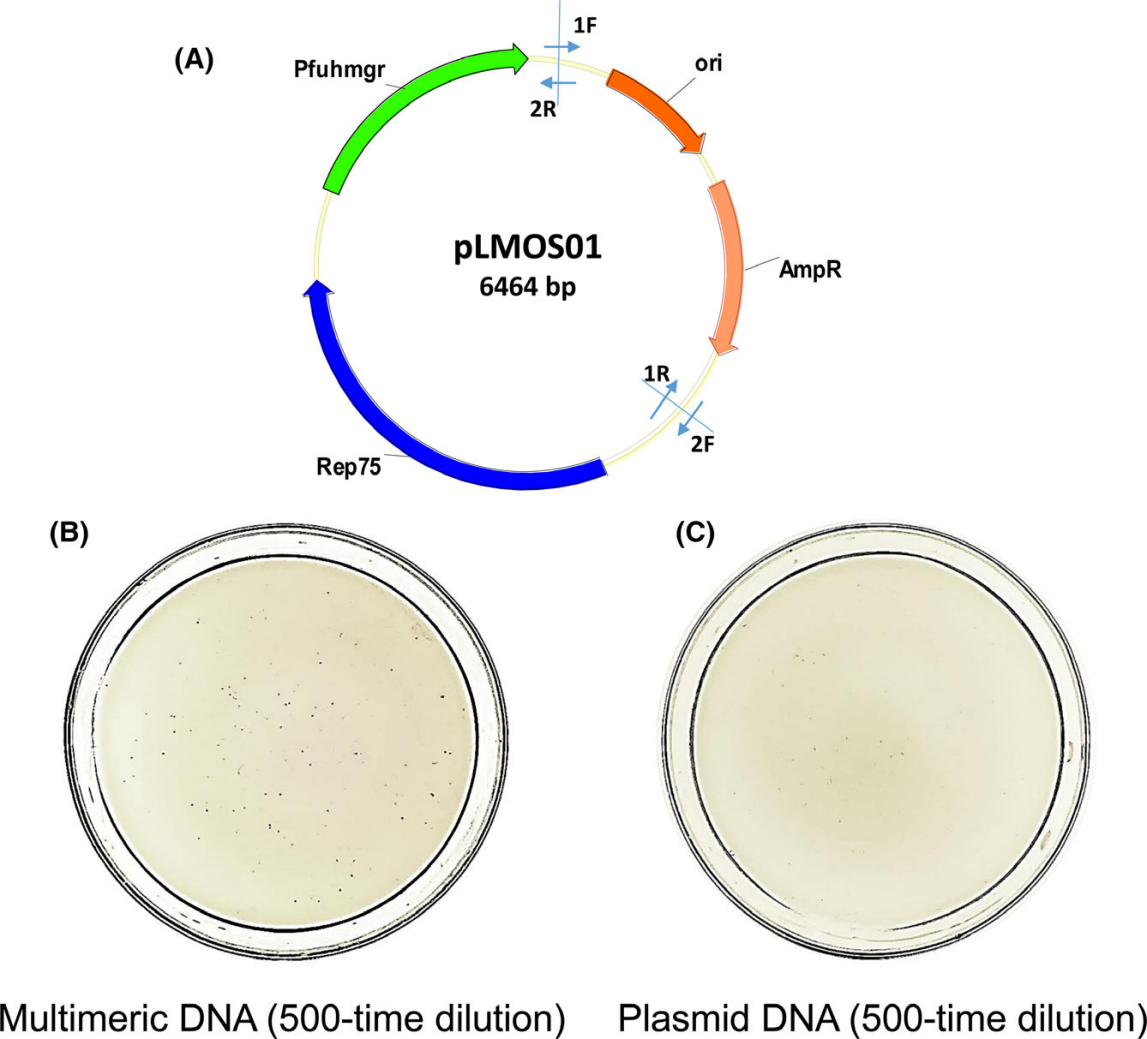
Comparison of *P. yayanosii* A1 transformation efficiency between multimeric DNA and plasmid DNA. A. Map of pLMOS01. Primers1F/1R and 2F/2R indicated were used to prepare multimeric DNA by POE‐PCR. Images of the transformants growing on the plates after the transformation of 1 μg of multimeric DNA. B. 1 μg of plasmid DNA. C. After 500 times dilution.

In addition, we tested whether the POE‐PCR product was a preferred genetic material for another hyperthermophilic archaeon *P. yayanosii* A1. Both *P. yayanosii* A1 and the *P. yayanosii*‐*E. coli* shuttle plasmid pLMOS01 were gifted from Prof. Xu (Fig. 6A) (Song et al., 2018a). It had the transformation efficiency of (6 ± 2) ×10^3^ transformants per μg of circular plasmid as reported in the literature (Song et al., 2018a). The transformation efficiency based on the POE‐PCR product was enhanced to (5 ± 1) × 10^4^ transformants per μg of DNA by a factor of 7.6 (Fig. 6B and C). These results suggested that direct transformation of POE‐PCR product was also applicable for *P. yayanosii* A1 (Fig. 6).

## Discussion

Although *T. kodakarensis* has a natural competence of the uptake and incorporation of exogenous DNA, the transformation efficiency of circular plasmid DNA was only about 10^2^ CFUs per μg of plasmid DNA (Hileman and Santangelo, 2012). In this study, we demonstrated that both archaea *T. kodakarensis* and *P. yayanosii* A1 preferred taking up multimeric DNA molecules (i.e. POE‐PCR products) to circular plasmids. The transformation efficiency of *T. kodakarensis* was enhanced up to 3 × 10^4^ CFUs per μg of multimeric DNA by a factor of 100 (Fig. 3). The similar increased transformation efficiency was also found in *P. yayanosii* A1 (Fig. 6). Also, we found that the transformation efficiency was related to the growth phase of the *T. kodakarensis* cells. Although multimeric DNA molecules have been used in the transformation of *E. coli*, *B. subtilis* and related *Bacillus* spp. (Zhang and Zhang, 2011; You *et al*., 2012; Liu *et al*., 2013; Cao *et al*., 2014; Fu *et al*., 2018), this study was the first example to demonstrate the enhanced transformation efficiencies of POE‐PCR products for archaea. This simple technology may work for other archaea. However, we had limited archaeal resources to test this technology for other archaea.

It was not known why these two archaea preferred taking up multimeric DNA molecules. We attempted to BLAST (basic local alignment search tool) to search homologues of several genes related to bacterial competences to published genomes of *T. kodakarensis* or *P. yayanosii* (Fukui *et al*., 2005; Claverys *et al*., 2009; Jun *et al*., 2011; Wagner *et al*., 2017). But we failed to find out any homologues, implying that archaea may have a different DNA uptake mechanism. The transformation mechanism of archaea may be worth further investigation. Such in‐depth understanding may help develop better genetic transformation techniques in the future.

Due to high‐efficiency transformation of archaea, a selection‐based strategy was demonstrated for direction evolution of *M. okinawensis* HMGR in *T. kodakarensis* in this study (Fig. 4B). Compared to the tedious steps in the routine method (Fig. 4A), this new strategy contains three steps: Step 1, making a linearized vector backbone by high‐fidelity PCR and a randomly mutated *Mohmgr* library by error‐prone PCR or other mutagenesis techniques, both of which contained 3′ and 5′ 40‐ to 50‐bp overlapping termini; Step 2, constructing a multimeric DNA library by POE‐PCR; and Step 3, conducting *T. kodakarensis* transformation and selection on solid plates (Fig. 4B). Only tens of microlitres products of POE‐PCR would be enough to have a large‐size mutant library of more than 10^4^ transformants. The new method does not need restriction enzymes or ligase and does not involve the preparation of a large amount of plasmid through the *E. coli* cell culture. Also, this technique not only is less laborious and time‐saving but also avoids possible DNA sequence bias between *E. coli* and archaea. Via this technology, a more thermostable HMGR mutant was obtained only through one‐round mutagenesis and selection (Fig. 5). The homology structure model of the mutant was built to explain the mechanism for its enhanced thermostability. HMGR is a dimer of homodimers, and the mutant of MoHMGR has three mutation sites (S287C, M322V and V379A) (Fig. S3A). All the three mutations were located far away from its catalytic site K239 (Vogeli *et al*., 2019). Two residues – S287C and M322V – are located between two subunits of the protein, while V379A is exposed on the protein surface (Fig. S3A). S287C may stabilize the protein by making hydrophobic interaction with aromatic ring of F295 (Fig. S3B). The replacement of a bulky residue V379 with alanine may stabilize protein by decreasing its solvent accessible surface area (Fig. S3C) (Ahmad *et al*., 2008; Zhou *et al*., 2018). M322V may be a neutral mutation because the interactions with the around residues did not change. Recent studies demonstrated that selection based on proper protein folding at high temperature in *Thermus thermophilus* was very efficient to obtain more thermostable variants (Chautard *et al*., 2007; Mate *et al*., 2019). Combined with this folding‐selection strategy, high transformation efficiency hyperthermophilic hosts, such as *T. kodakarensis*, would be more valuable to develop highly thermostable enzymes by directed evolution.

In conclusion, we developed a very simple technology of transforming the POE‐PCR products to archaea with high transformation efficiencies. This technique could not only help develop more hyperthermophilic enzyme mutants by directed evolution but also simplify genetical modification of archaea, which could be novel hosts for industrial biotechnology.

## Experimental procedures

### Chemicals

All chemicals were reagent grade or higher and were purchased from Sigma‐Aldrich (St. Louis, MO, USA), Solarbio (Beijing, China) or Sinopharm (Shanghai, China), unless otherwise noted. All enzymes for molecular biology experiments were purchased from New England BioLabs (NEB, Ipswich, MA, USA) or Takara (Shiga, Kusatsu, Japan).

### Strains and growth conditions

The strains used in this work are shown in Table S2. *E. coli* TOP10 was used for general DNA manipulation. *E. coli* strains were cultivated at 37°C in the Luria–Bertani (LB) medium containing 10 g l^−1^ tryptone, 5 g l^−1^ yeast extract and 10 g l^−1^ NaCl. Ampicillin was added to the medium at a concentration of 100 mg l^−1^. *T. kodakarensis* KOD1, and its derivatives were manipulated in an anaerobic chamber (Coy Lab Products, Michigan, USA) and cultivated in a nutrient‐rich medium ASW‐YT‐S^0^ under strictly anaerobic conditions at 85°C. The ASW‐YT medium contains 1× artificial sea water (ASW) (1 l of the ASW solution contains 20 g NaCl, 3 g MgCl_2_·6H_2_O, 6 g MgSO_4_·7H_2_O, 1 g (NH_4_)_2_SO_4_, 200 mg NaHCO_3_, 300 mg CaCl_2_·2H_2_O, 0.5 g KCl, 420 mg KH_2_PO_4_, 50 mg NaBr, 20 mg SrCl_2_·6H_2_O, and 10 mg Fe(NH_4_)_2_(SO_4_)_2_·6H_2_O); 0.5% (w/v) yeast extract; and 0.5% (w/v) tryptone supplemented with 1× trace mineral solution (1 l of 1000× contains 0.5 g MnSO_4_·H_2_O, 0.1 g CoCl_2_·6H_2_O, 0.1 g ZnSO_4_·7H_2_O, 0.01 g CuSO_4_·5H_2_O, 0.01 g AlK(SO_4_)_2_·12H_2_O, 0.01 g H_3_BO_3_, 0.01 g Na_2_MoO_4_·2H_2_O) and 1× vitamin mixture (1 l of 200× contains 0.2 g niacin, 0.08 g biotin, 0.2 g pantothenate, 0.2 g lipoic acid, 0.08 g folic acid, 0.2 g P‐aminobenzoic acid, 0.2 g thiamine, 0.2 g riboflavin, 0.2 g pyridoxine, and 0.2 g cobalamin) (Gehring *et al*., 2017). The ASW‐YT‐S^0^ medium was the ASW‐YT media supplemented with 0.2% (w/v) sulphur.

*Thermococcus kodakarensis* TS559 transformants growing without agmatine were selected on ASW‐YT plates (Song et al., 2018a,b), and *T. kodakarensis* transformants resistant to simvastatin (Sim) were selected on ASW‐YT plates containing simvastatin (Hileman and Santangelo, 2012). The solid media were prepared by adding 1% (w/v) Gelrite^®^ as described previously (Song et al., 2018a,b). Further modifications of the medium for selection of transformants were as described below. *P. yayanosii* A1 was cultivated in *Thermococcales* rich medium (TRM) under anaerobic conditions at 95°C (Li *et al*., 2015). The TRM medium contains (per litre of distilled water) 1 g yeast extract, 4 g tryptone, 3.3 g PIPES disodium salt, 23 g NaCl, 5 g MgCl_2_∙6H_2_O, 0.7 g KCl, 0.5 g (NH_4_)_2_SO_4_, 1 ml of 5% KH_2_PO_4_, 1 ml of 5% K_2_HPO_4_, 1 ml of 2% CaCl_2_∙2H_2_O, 0.05 g NaBr, 0.01 g SrCl_2_∙6H_2_O, 1 ml of 10 mM Na_2_WO_4_, 1 ml of 25 mM FeCl_3_, and 1 mg resazurin. The TRM medium was adjusted to pH 6.8 before autoclave. It was reduced with 0.5 g sodium sulphide and 10 g sulphur before use.

### Preparation of plasmids

All primers used for PCR are listed in Table S3. The *Tk*‐*Ec* shuttle vector pTE1 was constructed as described previously (Song et al., 2018a,b). In brief, two DNA fragments were amplified from plasmid pUC19 with primers 1F/1R and from pTS543 (Santangelo *et al*., 2010) with primers 2F/2R by high‐fidelity PCR. They were assembled by POE‐PCR, yielding plasmid pTE1 by Simple Cloning (You *et al*., 2012).

The gene (KEGG ID, Metok_1349) of *M. okinawensis*, encoding a 3‐hydroxy‐3‐methylglutaryl coenzyme A reductase (HMGR), was synthesized (Data S1) by GenScript (Nanjing, China). It was then subcloned into pET28a, yielding the plasmid pET28a‐*Mohmgr*. Two DNA fragments amplified from pET28a‐*Mohmgr* with primers Mohmgr‐1F/Mohmgr‐1R and from pLC70 with primers pLC70‐F/pLC70‐R, respectively, were used to make plasmid pLC70‐*Mohmgr* by Simple Cloning (You *et al*., 2012).

Approximately 1.5‐kbp DNA molecule (Sim^r^ cassette) containing *Mohmgr* transcribed from the constitutive *T. kodakarensis* P*gdh* promoter was cloned from pLC70‐*Mohmgr* into pTE1 to generate plasmid pTE‐*Mohmgr*. In brief, two DNA fragments amplified from plasmid pLC70‐*Mohmgr* with primers Mohmgr‐2F/Mohmgr‐2R and from pTE1 with primers pTE‐F/pTE‐R were used to generate plasmid pTE‐*Mohmgr* by Simple Cloning (You *et al*., 2012).

### Generation of Multimeric DNA by POE‐PCR

Multimeric DNA molecules were prepared by two steps. First, linear DNA fragments (i.e. inserted DNA fragment and vector backbone), both of which contained 3′ and 5′ 40‐ to 50‐bp overlapping termini, were generated by high‐fidelity PCR with the Takara PrimerSTAR Max DNA Polymerase. Second, the multimeric DNA molecules were generated based on these DNA templates by POE‐PCR. In POE‐PCR, each PCR tube contained 4 ng μl^−1^ purified insertion DNA fragment, equimolar purified vector backbone and PrimerSTAR Max Premix without the addition of primers, where the insertion and vector fragments were concomitantly used as primers and templates (Fig. 1). POE‐PCR was conducted as follows: denaturation at 98°C for 3 min, 25 to 30 cycles of denaturation at 98°C for 20 s, annealing at 65°C for 20 s, and extension at 72°C at a rate of 2 kb min^−1^ for Takara PrimerSTAR Max DNA Polymerase based on the length of the desired chimeric vector; extension at 72°C for 10 min.

### Transformation of *T. kodakarensis* KOD1

Genetic manipulation of *T. kodakarensis* was performed under anaerobic conditions. *T. kodakarensis* transformation was performed as described elsewhere (Sato *et al*., 2005) with minor modifications. Briefly, after cultivation of the host strain TS559 in ASW‐YT liquid medium supplemented with 1 mM agmatine, one mL of cell culture with an absorbance of 0.8 at 600 nm (i.e. ~ 8 × 10^8^ cells) was harvested in a 1.5‐ml microcentrifuge tube by centrifugation (6500 × *g*, 5 min), then resuspended in 200 μl of 0.8 × ASW medium, and kept on an ice bath for 30 min. One μl of different concentration of POE‐PCR product or purified plasmid DNA was added into the competent cells. The tubes containing cells were stood in an ice‐cold bath for 1 h, followed by a heat shock at 85°C for 45 s and incubation in an ice‐cold bath for 10 min. One ml of modified ASW‐YT liquid medium containing 2 ml of the polysulfide solution per litre was added to the transformed cells, and the competent cells were incubated at 85°C for 2 h. The cells were harvested by centrifugation (6500 × *g*, 5 min), resuspended in 200 μl of 0.8 × ASW, and then spread directly onto a solid ASW‐YT plate without agmatine or other selective conditions. After cultivation for 4–6 days at 85°C, the transformants growing on the plate were identified. Transformation efficiencies were determined by counting colony numbers. All values were averages of the results of three independent experiments. The DNA sequences of positive colonies identified by colony PCR were validated by DNA sequencing (GENEWIZ, Beijing, China) (Kanai *et al*., 2015).

### Transformation of *P. yayanosii* A1

Genetic manipulation of *P. yayanosii* A1 was performed under anaerobic conditions. *P. yayanosii* A1 transformation was performed as described elsewhere (Li *et al*., 2015) with minor modifications. The strain was cultivated in TRM medium at 95°C for 12 h. Fifteen ml of cells culture with an absorbance of 0.1 at 600 nm were collected by centrifugation (4000 r.p.m., 10 min) and resuspended in 200 μl of the transformation buffer (0.8 × ASW). The suspended cells were transferred into a 1.5‐ml microcentrifuge tube and then stood in an ice‐cold water bath for 0.5 h under anaerobic conditions. The POE‐PCR product (i.e. 5 μl of ~ 200 multimeric DNA ng μl^−1^) or purified plasmid DNA (5 μl of 200 ng DNA μl^−1^) was mixed gently with the competent cells and then kept in an ice‐cold water batch for 1 h. After a heat shock at 95°C for 45 s, the competent cells were kept in an ice‐cold water batch for 10 min. One millilitre of TRM medium (without simvastatin) was added to the transformed cells, and the cells were incubated at 95°C for 4 h. The diluted cell culture was spread onto the solid TRM medium supplemented with 10 μM simvastatin and then incubated for 4 ~ 6 days at 95°C. Transformation efficiencies were determined by counting colony numbers. All values were averages of the results of three independent experiments. The DNA sequences of positive colonies identified by colony PCR were validated by DNA sequencing (GENEWIZ, Beijing, China) (Kanai *et al*., 2015).

### Random mutagenesis by error‐prone PCR

A DNA mutant library encoding the *Mohmgr* gene was generated by low mutation rate error‐prone PCR with a pair of primers Mohmgr‐2F/Mohmgr‐2R. The reaction solution with a total volume of 50 μl contained 0.02 ng μl^−1^ plasmid pTE‐*Mohmgr*, 0.2 mM dATP, 0.2 mM dGTP, 1 mM dCTP, 1 mM dTTP, 5 mM MgCl_2_, 0.05 mM MnCl_2_, 0.05 U μl^−1^ NEB Taq polymerase, 0.4 mM Mohmgr‐2F and 0.4 mM Mohmgr‐2R. Error‐prone PCR was conducted as follows: 94°C denaturation for 2 min; 18 cycles of 94°C denaturation for 30 s, 55°C annealing for 30 s, 68°C extension for 70 s; and 68°C extension for 5 min. The linear pTE1 vector backbone was amplified with a pair of primers pTE‐F/pTE‐R by using the Takara PrimerSTAR Max DNA Polymerase. Then, the random mutagenesis libraries were constructed by POE‐PCR with Takara PrimerSTAR Max DNA Polymerase. The PCR products were introduced directly into *T. kodakarensis* TS559 cells and selected on the solid plates containing Sim.

### Structure analysis

The three‐dimensional structure modelling of wild‐type and mutant HMGR from *M. okinawensis* was built by SWISS‐MODEL (http://swissmodel.expasy.org/) based on the crystal structure of HMGR from *Methanothermococcus thermolithotrophicus* (PDB: 6HR8). The wild type of MoHMGR shared 83.4% sequence identity with the template of MtHMGR. The stereochemical quality of the homologue structure was checked by PROCHECK (http://services.mbi.ucla.edu/SAVES/). The interactions of residues were analysed by Discovery Studio 3.5 Client (Accelrys, San Diego, USA). The pictures were drawn by using Pymol (https://pymol.org/2/).

### Other assays

All the DNA concentrations were detected by an Eppendorf BioSpectrometer^®^ equipment with an Eppendorf µCuvette.

## Conflict of interest

None declared.

## Supporting information


**Table S1**. Comparison of transformation methods for a number of hyperthermophilic archaea.
**Table S2**. Strains and plasmids used in this study.
**Table S3**. Primers used in this study.
**Fig. S1**. Colony PCR validation of the multimeric DNA transformants.
**Fig. S2**. Preparation of the *Mohmgr* mutant library.
**Fig. S3**. Structure analysis of the MoHMGR mutant M1.
**Data S1**. Supplementary DNA sequence 1 of the codon optimized *Mohmgr*.
**Data S2**. Supplementary DNA sequence 2 of the *Mohmgr* mutant M1.
**Data S3**. Supplementary amino acid sequence 3 of the MoHMGR mutant M1.
