# Supplementary material for: High‐efficiency transformation of archaea by direct PCR products with its application to directed evolution of a thermostable enzyme
Source: Microb Biotechnol. 2020 Jun 29;14(2):453–64. doi: 10.1111/1751-7915.13613 (PMC7936305; doi:10.1111/1751-7915.13613)
Supplement: Supplementary file 1 — Table S1. Comparison of transformation methods for a number of hyperthermophilic archaea. Table S2. Strains and plasmids used in this study. Table S3. Primers used in this study. Fig. S1. Colony PCR validation of the multimeric DNA transformants. Fig. S2. Preparation of the Mohmgr mutant library. Fig. S3. Structure analysis of the MoHMGR mutant M1. Data S1. Supplementary DNA sequence 1 of the codon optimized Mohmgr. Data S2. Supplementary DNA sequence 2 of the Mohmgr mutant M1. Data S3. Supplementary amino acid sequence 3 of the MoHMGR mutant M1. [file MBT2-14-453-s001.docx]

**Supplementary Data**

High efficiency transformation of archaea by direct PCR products with its application to directed evolution of a thermostable enzyme

Yunhong Song^1^ Zhiguang Zhu^1^ Wei Zhou^1^ Yi-Heng P. Job Zhang^1*^

^1^ Tianjin Institute of Industrial Biotechnology, Chinese Academy of Sciences, 32 West 7th Avenue, Tianjin Airport Economic Area, Tianjin 300308, China

* To whom correspondence should be addressed. Yi-Heng P. Job Zhang (ORCID ID: 0000-0002-4010-2250), Tel: 001-540-449-1190, email: yhjob_zhang@outlook.com

# Table S1. Transformation methods for hyperthermophilic archaea.

| **Organism** | **T_opt_** | **Genetic modification** | **Vector** | **Transformation method** | **Selection** | **Counter-selection** | **Transformation efficiency**  **(CFU/ ug DNA)** | **References** |
| --- | --- | --- | --- | --- | --- | --- | --- | --- |
| *Pyrococcus abyssi* | 100 | Replicating vector | Plasmid | PEG/Spheroplast | -Ura | 5FOA | 10^2^ | ^1^ |
| *Pyrococcus yayanosii* | 98 | Homologous  recombination | Plasmid | Natural competence, CaCl_2_ +heat shock | Sim, -Ura | 5FOA | 10^2^ | ^2^ |
|  |  | Replicating vector | Plasmid | Natural competence, CaCl_2_ +heat shock |  |  | 6.3×10^3^ | This study |
|  |  | Replicating vector | Multimeric DNA |  | Sim |  | 4.8×10^4^ | This study |
| *Pyrococcus* *furiosus* | 100 | Homologous  recombination | Plasmid, BAC, or linear DNA | Natural competence | -Ura, | 5FOA, 6MP | 10^4-^10^5^ | ^3-5^ |
|  |  | Replicating vector | Plasmid | Natural competence, heat shock | Sim |  | 10^2^ | ^6^ |
| *Sulfolobus acidocaldarius* | 80 | Homologous  recombination | Plasmid or linear DNA | Electroporation | -Ura | 5FOA | 10^3-^10^4^ | ^7-9^ |
|  |  | Replicating vector | Plasmid | Electroporation | -Ura, -Agm |  | 10^2-^10^4^ | ^9,10^ |
| *Sulfolobus islandicus* | 78 | Homologous recombination | Plasmid or linear DNA | Electroporation | -Ura, Sim, -Agm | 5FOA | NR | ^11-14^ |
|  |  | Replicating vector | Plasmid | Electroporation | -Ura, Sim, -Agm |  | 10^2-^10^4^ | ^11,13-15^ |
| *Sulfolobus solfataricus* | 80 | Homologous  recombination | Plasmid or linear DNA | Electroporation | Lactose, -Ura | NR | NR | ^16,17^ |
|  |  | Replicating vector | Plasmid | Electroporation | Lactose, butanol/benzyl alcohol, HygB |  | 10^2^-10^4^ | ^18-20^ |
| *Thermococcus kodakarensis* | 85 | Homologous  recombination | Plasmid or linear DNA | Natural competence, CaCl_2_ +heat shock | -Ura, -Trp, Sim | 5FOA, 6MP | 10^1^-10^2^ | ^21-24^ |
|  |  | Replicating vector | Plasmid |  | -Trp, Mev, -Agm |  | 10^0^-10^2^ | ^24,25^ |
|  |  | Replicating vector | Multimeric DNA | Natural competence, CaCl_2_ +heat shock | Mev, -Agm |  | 3×10^4^ | This study |
| *Thermococcus barophilus* | 85 | Homologous recombination | Plasmid | Heat shock | Sim | 5FOA | 10^2^ | ^26,27^ |
| *Thermococcus* *onnurineus* | 80 | Homologous recombination | Plasmid | CaCl_2_ +heat shock | Sim | NR | NR | ^28^ |
| *Methanocaldococcus jannaschii* | 80 | Homologous recombination | Linear plasmid | Heat shock | Sim, Mev | NR | 10^3^-10^4^ | ^29^ |

NR, not reported; BAC, bacterial artificial chromosome; 5FOA, 5-fluoroorotic acid; 6MP, 6-methylpurine; Agm, agmatine; Mev, mevinolin; Sim, simvastatin; Trp, tryptophan; and Ura, uracil

# Table S2. Strains and plasmids used in this study.

| Strains or plasmids | Genetic features | Source or reference |
| --- | --- | --- |
| Strains |  |  |
| Top 10 | F^-^ *mcrA Δ(mrr-hsd*RMS*-mcr*BC*) φ80lac*Z*ΔM15 Δlac*X*74 nup*G *rec*A*1 ara*D*139 Δ(ara-leu)7697 gal*E*15 gal*K*16 rps*L(Str^R^) *end*A*1 λ^-^* | Invitrogen,  Carlsbad, CA |
| TS559 | *T. kodakarensis* △*pyr*F;△*trp*E::*pyr*F;△TK0149 | ^24^ |
| KOD1 | *T. kodakarensis* wild type strain | ^30^ |
| *P.yayanosii* A1 | A facultatively piezophilic derivative strain of the wild-type *P.yayanosii CH1* | ^2^ |
| Plasmids |  |  |
| pTE1 | Tk*pdaD*, Amp^r^ | ^31^ |
| pLC70 | P_gdh_-PF1848, Amp^r^, Kan^r^ | ^25^ |
| pLC70-*Mohmgr* | P_gdh_-Mohmgr, Amp^r^ | This study |
| pTE-*Mohmgr* | P_gdh_-Mo*hmgr*, Tk*pdaD*, Amp^r^ | This study |
| pTE-*Mohmgr*M1 | Thermostable Mo*hmgr* mutant 1, Tk*pdaD*, Amp^r^ | This study |
| pLMOS01 | Shuttle plasmid replicated in both *P.yayanosii* and *E. coli* | ^32^ |

# Table S3. Primers used in this study

| Primers | Sequences (5’→3’) | Source |
| --- | --- | --- |
| 1F | CAATTTCACACAGGAAACAGCTATGGCCAATTCTGGCAATTTGGCAAATTC | ^31^ |
| 1R | GCCACCTGACGTCTAAGAAACCATTGTGGAGTGAAGTACCACCTGAGCTTC | ^31^ |
| 2F | GAAGCTCAGGTGGTACTTCACTCCACAATGGTTTCTTAGACGTCAGGTGGC | ^31^ |
| 2R | GAATTTGCCAAATTGCCAGAATTGGCCATAGCTGTTTCCTGTGTGAAATTG | ^31^ |
| Mohmgr-1F | CTAAATTACTGCAGAGGTGGTATGAATGGAGGACATAACGAAGATAGTCG | This study |
| Mohmgr-1R | CTGCAGATATCCATATCGGATCCTCACCTTCCGAGCTCGCTGTGGGCCTTG | This study |
| pLC70-F | CAAGGCCCACAGCGAGCTCGGAAGGTGAGGATCCGATATGGATATCTGCAG | This study |
| pLC70-R | CGACTATCTTCGTTATGTCCTCCATTCATACCACCTCTGCAGTAATTTAG | This study |
| Mohmgr-2F | CGGCAGTCGACTTTTTTGCGGCCGCTCTAGATGAACTTTGATGAACGTAAAAG | This study |
| Mohmgr-2R | CTGCAGATATCCATATCGGATCCTCACCTTCCGAGCTCGCTGTGGGCCTTG | This study |
| pTE-F | CAAGGCCCACAGCGAGCTCGGAAGGTGAGGATCCGATATGGATATCTGCAG | This study |
| pTE-R | CTTTTACGTTCATCAAAGTTCATCTAGAGCGGCCGCAAAAAAGTCGACTGCCG | This study |
| pLMOS01-1F | GTCACGACGTTGTAAAACGACGGCCAGTGCCAAGCTTGCATGCCTGCAGG | This study |
| pLMOS01-1R | GTGAGCGGATAACAATTTCACACAGGAAACAGCTATGACCATGATTACG | This study |
| pLMOS01-2F | CGTAATCATGGTCATAGCTGTTTCCTGTGTGAAATTGTTATCCGCTCAC | This study |
| pLMOS01-2R | CCTGCAGGCATGCAAGCTTGGCACTGGCCGTCGTTTTACAACGTCGTGAC | This study |


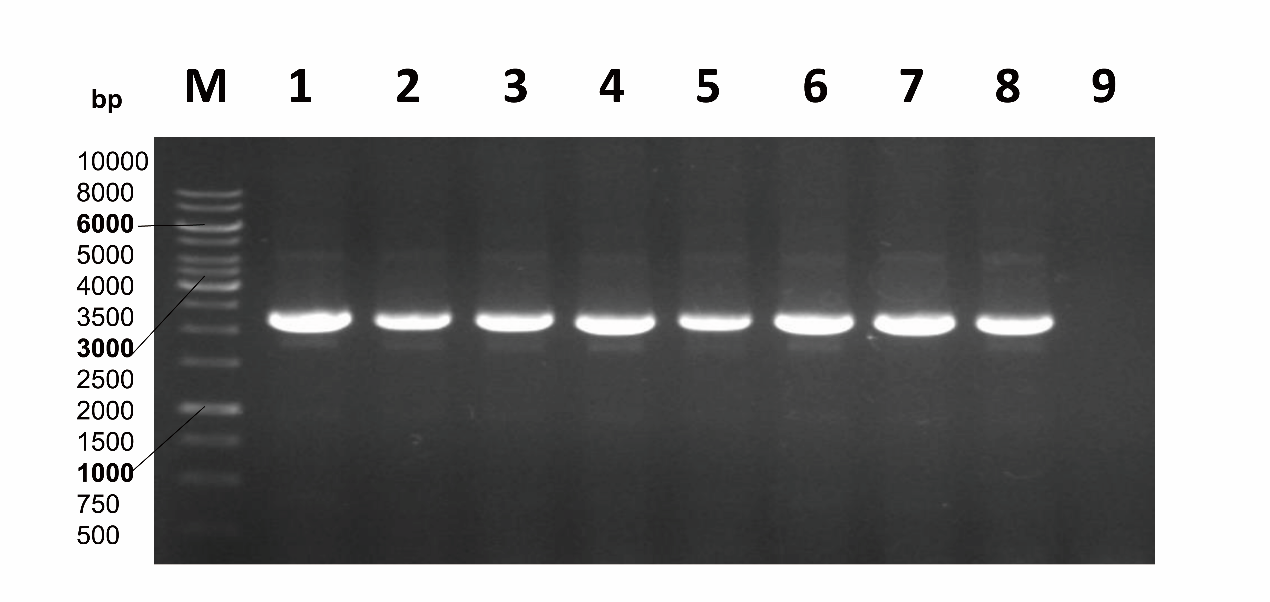


# Figure S1.

Colony PCR validation of the multimeric DNA transformants. M: a 1 kb DNA ladder from Thermo Scientific, lane 1-7, transformants of POE-PCR products, lane 8 transformant of plasmid DNA, lane 9: empty host. Primers 1F/1R were used to check the shuttle plasmid, the correct PCR products is about 2200 bp .


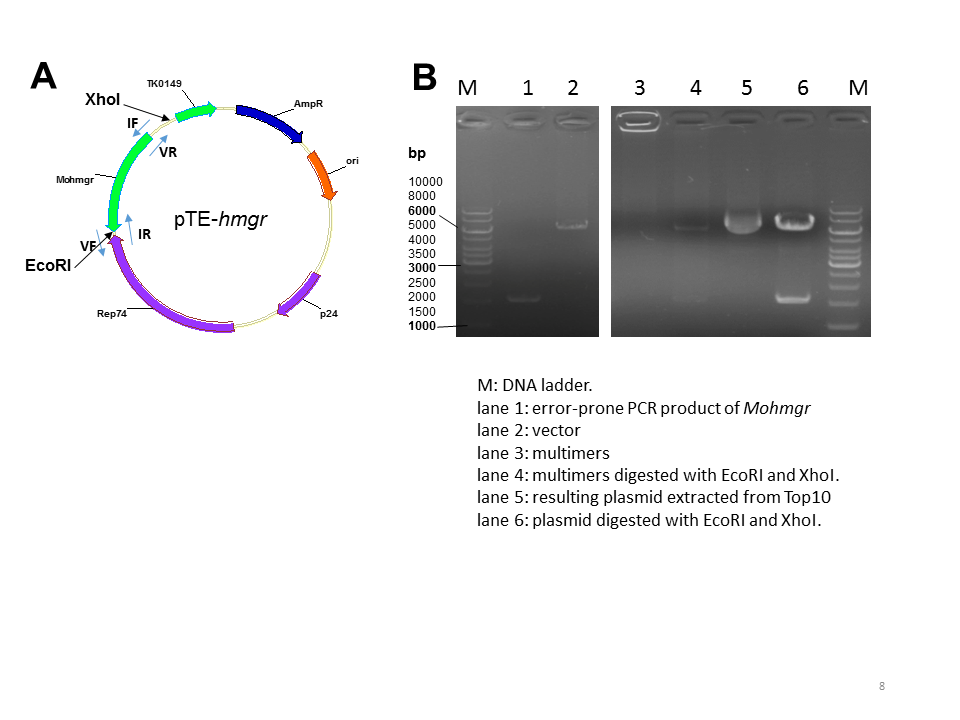


# Figure S2.

Preparation of Mohmgr mutant library. (**A**) Map of pTE-Mohmgr. (**B**) Agarose analysis of DNA mutant library and resulting plasmid.


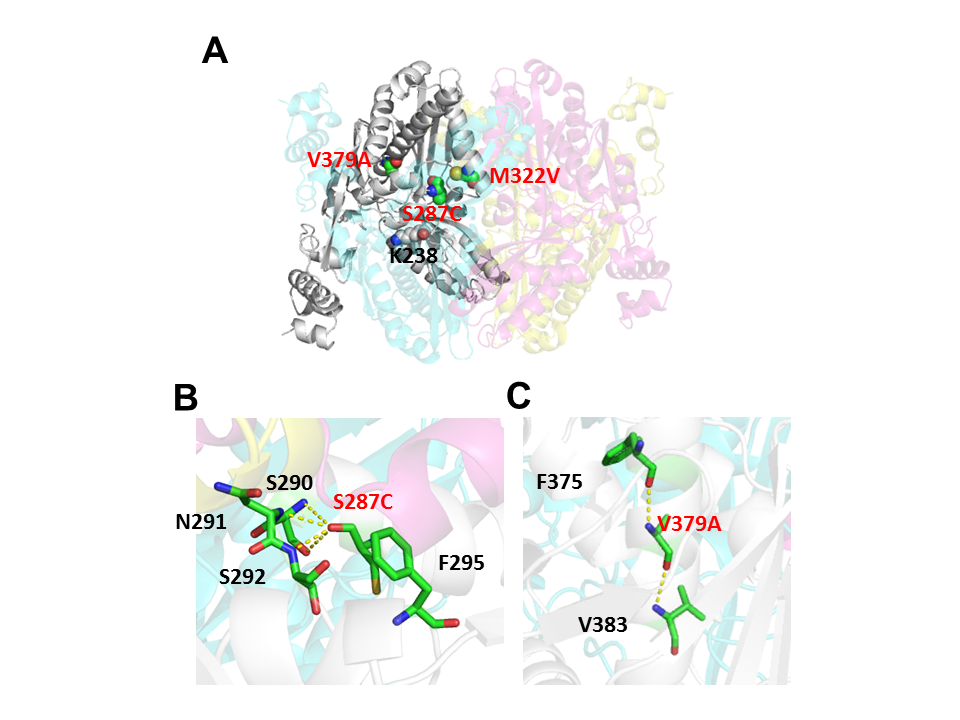


# Figure S3.

Structure analysis of MoHMGR mutant M1. A. The distribution of the mutation sites (S287C, M322V, V379A) and the catalytic site K238, where mutation and catalytic residues mutant are indicated in sphere representation. HMGR is a dimer of homodimers, and subunits are indicated in different colors. B&C. Local environment of mutation sites S287C and V379A.

# Supplementary DNA sequence 1

> codon optimized Mo*hmgr*

atggaggacataacgaagatagtcgagaagctcaagaacaaggagataaagccgtaccagctcgacaacatgttcgacagcaagaagagcgttgagataaggagaaagtacatagagagcctcacggacaccgccttcaagcacatagacaagtacagcataaacgagaaggaagccatggagaagaacatagagaacatgataggcgccgtccagataccgctcggattcgccggccccctcaagataaacggaaagtacgccaacggcgagtactacatacccctcgccaccaccgagggagccctcgttgccagcgtcaacaggggatgcggcataataacgaagtgcggcggatgcaccgtcagggttgtcgacgacaagatgacgagggcccccgttataaagaccaacagcataatagacgccataaagctcaaggagtggatagagaacaacttccagaagataaaggaagttgccgagaccacgaccaagcacggaaagctcataagcataaacccgataataatagttggcagatacgtctaccccaggttcacgtacaagaccggagacgccatgggcatgaacatggtcacgatagccaccgagaaggcctgcaacttcatagagaacgagatgaagaaggaagagaacggcagcataaaggttcacacggtcgccctcagcggaaacgtttgcaccgacaagaagcccgccggcataaacctcatagagggaaggggcaagaccataatagccgaggtcttcctcaaggaagaggagataaagaagtacctcaagacgaccagcaaggccatagagcaggttaacatgtacaagaacttcataggaagcgccataagcaacagcatgggcttcaacgcccactacgccaacataataggagccctcttcctcgccaccggacaggacgaggcccacatagtcgagggaagcatgggcataaccgttgccgagtgcgtcgacgacggactctacttcagcgttacgctccccgacgtcccaataggaacggttggcggaggcacgagggttgagacccagaaggagtgcctggagatgataggatgcaggggaggcgacaaggccctcaagttcgccgagatagttggaggcgccgtcctcgccggagagctcagcctcatgggagccctcgccgccggacacctcgccaaggcccacagcgagctcggaaggctcgagcaccaccaccaccaccactga

# Supplementary DNA sequence 2

> Mo*hmgr* mutant M1

atggaggacataacgaagatagtcgagaagctcaagaacaaggagataaagccgtaccagctcgacaacatgttcgacagcaagaagagcgttgagataaggagaaagtacatagagagcctcacggacaccgccttcaagcacatagacaagtacagcataaacgagaaggaagccatggagaagaacatagagaacatgataggcgccgtccagataccgctcggattcgccggccccctcaagataaacggaaagtacgccaacggcgagtactacatacccctcgccaccaccgagggagccctcgttgccagcgtcaacaggggatgcggcataataacgaagtgcggcggatgcaccgtcagggttgtcgacgacaagatgacgagggcccccgttataaagaccaacagcataatagacgccataaagctcaaggagtggatagagaacaacttccagaagataaaggaagttgccgagaccacgaccaagcacggaaagctcataagcataaacccgataataatagttggcagatacgtctaccccaggttcacgtacaagaccggagacgccatgggcatgaacatggtcacgatagccaccgagaaggcctgcaacttcatagagaacgagatgaagaaggaagagaacggcagcataaaggttcacacggtcgccctcagcggaaacgtttgcaccgacaagaagcccgccggcataaacctcatagagggaaggggcaagaccataatagccgaggtcttcctcaaggaagaggagataaagaagtacctcaagacgaccagcaaggccatagagcaggttaacatgtacaagaacttcataggatgcgccataagcaacagcatgggcttcaacgcccactacgccaacataataggagccctcttcctcgccaccggacaggacgaggcccacatagtcgagggaagcgtgggcataaccgttgccgagtgcgtcgacgacggactctacttcagcgttacgctccccgacgtcccaataggaacggttggcggaggcacgagggttgagacccagaaggagtgcctggagatgataggatgcaggggaggcgacaaggccctcaagttcgccgagatagctggaggcgccgtcctcgccggagagctcagcctcatgggagccctcgccgccggacacctcgccaaggcccacagcgagctcggaaggtga

# Supplementary amino acid sequence 3

> Mo*hmgr* mutant M1

MEDITKIVEKLKNKEIKPYQLDNMFDSKKSVEIRRKYIESLTDTAFKHIDKYSINEKEAMEKNIENMIGAVQIPLGFAGPLKINGKYANGEYYIPLATTEGALVASVNRGCGIITKCGGCTVRVVDDKMTRAPVIKTNSIIDAIKLKEWIENNFQKIKEVAETTTKHGKLISINPIIIVGRYVYPRFTYKTGDAMGMNMVTIATEKACNFIENEMKKEENGSIKVHTVALSGNVCTDKKPAGINLIEGRGKTIIAEVFLKEEEIKKYLKTTSKAIEQVNMYKNFIGCAISNSMGFNAHYANIIGALFLATGQDEAHIVEGSVGITVAECVDDGLYFSVTLPDVPIGTVGGGTRVETQKECLEMIGCRGGDKALKFAEIAGGAVLAGELSLMGALAAGHLAKAHSELGR

# References

1. Lucas, S., Toffin, L., Zivanovic, Y., Charlier, D., Moussard, H., Forterre, P., Prieur, D. and Erauso, G. (2002) Construction of a shuttle vector for, and spheroplast transformation of, the hyperthermophilic archaeon *Pyrococcus abyssi*. *Appl. Environ. Microbiol.*, **68**, 5528-5536.

2. Li, X., Fu, L., Li, Z., Ma, X., Xiao, X. and Xu, J. (2015) Genetic tools for the piezophilic hyperthermophilic archaeon *Pyrococcus yayanosii*. *Extremophiles*, **19**, 59.

3. Lipscomb, G.L., Stirrett, K., Schut, G.J., Yang, F., Jenney, F.E., Scott, R.A., Adams, M.W.W. and Westpheling, J. (2011) Natural competence in the hyperthermophilic archaeon *Pyrococcus furiosus* facilitates genetic manipulation: construction of markerless deletions of genes encoding the two cytoplasmic hydrogenases. *Appl. Environ. Microbiol.*, **77**, 2232-2238.

4. Farkas, J., Stirrett, K., Lipscomb, G.L., Nixon, W., Scott, R.A., Adams, M.W. and Westpheling, J. (2012) Recombinogenic properties of *Pyrococcus furiosus* strain COM1 enable rapid selection of targeted mutants. *Appl. Environ. Microbiol.*, **78**, 4669-4676.

5. Kreuzer, M., Schmutzler, K., Waege, I., Thomm, M. and Hausner, W. (2013) Genetic engineering of *Pyrococcus furiosus* to use chitin as a carbon source. *BMC Biotechnol.*, **13**, 9.

6. Waege, I., Schmid, G., Thumann, S., Thomm, M. and Hausner, W. (2010) Shuttle vector-based transformation system for *Pyrococcus furiosus*. *Appl. Environ. Microbiol.*, **76**, 3308-3313.

7. Sakofsky, C.J., Runck, L.A. and Grogan, D.W. (2011) *Sulfolobus* mutants, generated via PCR products, which lack putative enzymes of UV photoproduct repair. *Archaea*, **2011**, 864015.

8. Wagner, M., van Wolferen, M., Wagner, A., Lassak, K., Meyer, B.H., Reimann, J. and Albers, S.V. (2012) Versatile genetic tool box for the crenarchaeote *Sulfolobus acidocaldarius*. *Front. Microbiol.*, **3**, 214.

9. Suzuki, S. and Kurosawa, N. (2017) Development of the multiple gene knockout system with one-step PCR in thermoacidophilic crenarchaeon *Sulfolobus acidocaldarius*. *Archaea*, **2017**, 7459310.

10. Berkner, S., Grogan, D., Albers, S.V. and Lipps, G. (2007) Small multicopy, non-integrative shuttle vectors based on the plasmid pRN1 for *Sulfolobus acidocaldarius* and *Sulfolobus solfataricus*, model organisms of the (cren-)archaea. *Nucleic. Acids. Res.*, **35**, e88.

11. Deng, L., Zhu, H., Chen, Z., Liang, Y.X. and She, Q. (2009) Unmarked gene deletion and host-vector system for the hyperthermophilic crenarchaeon *Sulfolobus islandicus*. *Extremophiles*, **13**, 735-746.

12. Zhang, C. and Whitaker, R.J. (2012) A broadly applicable gene knockout system for the thermoacidophilic archaeon *Sulfolobus islandicus* based on simvastatin selection. *Microbiology*, **158**, 1513-1522.

13. Jaubert, C., Danioux, C., Oberto, J., Cortez, D., Bize, A., Krupovic, M., She, Q., Forterre, P., Prangishvili, D. and Sezonov, G. (2013) Genomics and genetics of *Sulfolobus islandicus* LAL14/1, a model hyperthermophilic archaeon. *Open Biol.*, **3**, 130010.

14. Zhang, C., Cooper, T.E., Krause, D.J. and Whitaker, R.J. (2013) Augmenting the genetic toolbox for *Sulfolobus islandicus* with a stringent positive selectable marker for agmatine prototrophy. *Appl. Environ. Microbiol.*, **79**, 5539-5549.

15. Zheng, T., Huang, Q., Zhang, C., Ni, J., She, Q. and Shen, Y. (2012) Development of a simvastatin selection marker for a hyperthermophilic acidophile, *Sulfolobus islandicus*. *Appl. Environ. Microbiol.*, **78**, 568-574.

16. Worthington, P., Hoang, V., Perez-Pomares, F. and Blum, P. (2003) Targeted disruption of the  α-amylase gene in the hyperthermophilic archaeon *Sulfolobus solfataricus*. *J. Bacteriol.*, **185**, 482-488.

17. Jonuscheit, M., Martusewitsch, E., Stedman, K.M. and Schleper, C. (2003) A reporter gene system for the hyperthermophilic archaeon *Sulfolobus solfataricus* based on a selectable and integrative shuttle vector. *Mol. Microbiol.*, **48**, 1241-1252.

18. Cannio, R., Contursi, P., Rossi, M. and Bartolucci, S. (1998) An autonomously replicating transforming vector for *Sulfolobus solfataricus*. *J. Bacteriol.*, **180**, 3237-3240.

19. Contursi, P., Cannio, R., Prato, S., Fiorentino, G., Rossi, M. and Bartolucci, S. (2003) Development of a genetic system for hyperthermophilic archaea: expression of a moderate thermophilic bacterial alcohol dehydrogenase gene in *Sulfolobus solfataricus*. *FEMS Microbiol. Lett.*, **218**, 115-120.

20. Albers, S.-V. and Driessen, A.J.M. (2008) Conditions for gene disruption by homologous recombination of exogenous DNA into the *Sulfolobus solfataricus* genome. *Archaea*, **2**, 145-149.

21. Sato, T., Fukui, T., Atomi, H. and Imanaka, T. (2003) Targeted gene disruption by homologous recombination in the hyperthermophilic archaeon *Thermococcus kodakaraensis* KOD1. *J. Bacteriol.*, **185**, 210-220.

22. Sato, T., Fukui, T., Atomi, H. and Imanaka, T. (2005) Improved and versatile transformation system allowing multiple genetic manipulations of the hyperthermophilic archaeon *Thermococcus kodakaraensis*. *Appl. Environ. Microbiol.*, **71**, 3889-3899.

23. Matsumi, R., Manabe, K., Fukui, T., Atomi, H. and Imanaka, T. (2007) Disruption of a sugar transporter gene cluster in a hyperthermophilic archaeon using a host-marker system based on antibiotic resistance. *J. Bacteriol.*, **189**, 2683-2691.

24. Santangelo, T.J., Čuboňová, L.u. and Reeve, J.N. (2010) *Thermococcus kodakarensis* genetics: TK1827-encoded β-glycosidase, new positive-selection protocol, and targeted and repetitive deletion technology. *Appl. Environ. Microbiol.*, **76**, 1044-1052.

25. Santangelo, T.J., Čuboňová, L.u. and Reeve, J.N. (2008) Shuttle vector expression in *Thermococcus kodakaraensis*: contributions of *cis* elements to protein synthesis in a hyperthermophilic archaeon. *Appl. Environ. Microbiol.*, **74**, 3099-3104.

26. Thiel, A., Michoud, G., Moalic, Y., Flament, D. and Jebbar, M. (2014) Genetic manipulations of the hyperthermophilic piezophilicarchaeon *Thermococcus barophilus*. *Appl. Environ. Microbiol.*, **80**, 2299-2306.

27. Cario, A., Mizgier, A., Thiel, A., Jebbar, M. and Oger, P.M. (2015) Restoration of the di-myo-inositol-phosphate pathway in the piezo-hyperthermophilic archaeon *Thermococcus barophilus*. *Biochimie*, **118**, 286-293.

28. Kim, M.-S., Bae, S.S., Kim, Y.J., Kim, T.W., Lim, J.K., Lee, S.H., Choi, A.R., Jeon, J.H., Lee, J.-H., Lee, H.S. *et al.* (2013) CO-dependent H_2_ production by genetically engineered *Thermococcus onnurineus* NA1. *Appl. Environ. Microbiol.*, **79**, 2048-2053.

29. Susanti, D., Frazier, M.C. and Mukhopadhyay, B. (2019) A genetic system for *Methanocaldococcus jannaschii*: An evolutionary deeply rooted hyperthermophilic methanarchaeon. *Front. Microbiol.*, **10**, 1256.

30. Atomi, H., Fukui, T., Kanai, T., Morikawa, M. and Imanaka, T. (2004) Description of *Thermococcus kodakaraensis* sp. nov., a well studied hyperthermophilic archaeon previously reported as *Pyrococcus* sp. KOD1. *Archaea*, **1**, 263-267.

31. Song, Y., Liu, M., Xie, L., You, C., Sun, J. and Zhang, Y.-H.P.J. (2018) A Recombinant 12-his tagged *Pyrococcus furiosus* soluble [NiFe]-hydrogenase I overexpressed in *Thermococcus kodakarensis* KOD1 facilitates hydrogen-powered *in vitro* NADH regeneration. *Biotechnol. J.*, **14**, e1800301.

32. Song, Q., Li, Z., Chen, R., Ma, X., Xiao, X. and Xu, J. (2018) Induction of a toxin-antitoxin gene cassette under high hydrostatic pressure enables markerless gene disruption in the hyperthermophilic archaeon *Pyrococcus yayanosii*. *Appl. Environ. Microbiol.*, **85**, e02662-02618.
